# Improved ORB Algorithm Using Three-Patch Method and Local Gray Difference

**DOI:** 10.3390/s20040975

**Published:** 2020-02-12

**Authors:** Chaoqun Ma, Xiaoguang Hu, Jin Xiao, Huanchao Du, Guofeng Zhang

**Affiliations:** State Key Laboratory of Virtual Reality Technology and Systems, School of Automation Science and Electrical Engineering, Beihang University, Beijing 100191, China; machaoqun0204@126.com (C.M.); xiaoguang@buaa.edu.cn (X.H.); duhuanchao@gmail.com (H.D.); gfzhang@buaa.edu.cn (G.Z.)

**Keywords:** image registration, three-patch method, local gray difference, feature fusion

## Abstract

This paper presents an improved Oriented Features from Accelerated Segment Test (FAST) and Rotated BRIEF (ORB) algorithm named ORB using three-patch and local gray difference (ORB-TPLGD). ORB takes a breakthrough in real-time aspect. However, subtle changes of the image may greatly affect its final binary description. In this paper, the feature description generation is focused. On one hand, instead of pixel patch pairs comparison method used in present ORB algorithm, a three-pixel patch group comparison method is adopted to generate the binary string. In each group, the gray value of the main patch is compared with that of the other two companion patches to determine the corresponding bit of the binary description. On the other hand, the present ORB algorithm simply uses the gray size comparison between pixel patch pairs, while ignoring the information of the gray difference value. In this paper, another binary string based on the gray difference information mentioned above is generated. Finally, the feature fusion method is adopted to combine the binary strings generated in the above two steps to generate a new feature description. Experiment results indicate that our improved ORB algorithm can achieve greater performance than ORB and some other related algorithms.

## 1. Introduction

Image registration, as an important issue in the computer vision field, is defined as the establishment of correspondences between two or more images of the same scene taken at different time, from different viewpoints. It is a fundamental process that is widely used in a variety of applications such as image matching [1,2,3,4,5,6], change detection [7,8], 3D reconstruction, and mapping sciences [9,10,11,12,13,14]. The matching of remote sensing images is studied by [1,6]. Automatic image matching is mentioned in [2]. The optimization of image matching process and parameters has been the researched in [3,4]. A probabilistic neural-network-based feature-matching algorithm for a stereo image pair is presented in [5]. A new approach for the registration of optical imagery with Light Detection and Ranging (LiDAR) data based on the theory of mutual information (MI) is proposed in [9]. 3D stereo matching is studied by [10,11]. Building recognition and extraction from images have been the researched in [12,13]. An automated feature matching method for Volunteer Geographic Information (VGI) linear data is presented in [14]. During the last decades, a variety of methods have been developed for image registration.

Currently, most image matching algorithms are based on feature-based methods because they are robust to geometric and illumination differences [15]. Feature-based methods are often expressed as a point-matching problem because representations of points are generic and easy to extract. This paper also focuses on point-based methods. The image matching algorithms based on feature-based methods generally include the following three main steps as follows:

1. Feature detection: extracting distinctive image features, such as corners, spots, and regions, from the given images (for example, a reference image and an input image).

2. Feature description: using various image attributes (such as grayscale gradients, textures, and edges) for providing a powerful representation of each the extracted local features in order to characterize and match the extracted local features.

3. Feature matching: establishing the correspondence of the extracted features from two images by using particular similarity measures [16].

Feature-based methods can be divided into two categories according to the different representation types of feature description: floating-point feature-based description method and binary feature-based description method.

One of the most famous feature extraction algorithms based on floating-point descriptors methods is the Scale Invariant Feature Transform (SIFT) [17] method proposed by Lowe in 1999 and improved in 2004. The SIFT algorithm theoretically has complete scale invariance and has good anti-interference ability for illumination, noise, rotation, zoom, occlusion, and even affine transformation. However, SIFT puts a heavy burden on the calculation amount. The feature point detection based on scale space extremum and the feature description based on gradient histogram severely restricts the computational efficiency of SIFT. This shortcoming has caused extensive efforts to optimize its speed without compromising its quality too much.

On the other hand, binary descriptors were designed with an emphasis on minimizing computational and storage costs [18,19,20,21,22,23]. Two commonly used binary feature description methods, Fast Retina Keypoint (FREAK) and Binary Robust Invariant Scalable Keypoints (BRISK), are proposed by [18,19]. The boosting is adopted in [20,21,22] to enhance algorithm performance. The descriptor vectors are mapped into the Hamming space in which the Hamming metric is used to reduce the size of the descriptors [23]. Image patches are projected to a more discriminative subspace, and threshold their coordinates to build a binary descriptor to enhance recognition performances [24]. These methods describe a feature point using a binary string. Though the binary description may not be as descriptive as floating-point method, they make up for this shortcoming in their compact size, efficient computation, and the ability to quickly compare descriptor pairs using few processor-level instructions. Binary Robust Independent Elementary Features (BRIEF) [6] is based on intensity comparisons of random pixel pairs in a patch centered around the feature point. These comparisons result in binary strings that can be matched very quickly using a simple exclusive OR (XOR) operation. As BRIEF is based on intensity comparisons, instead of image gradient computations and histogram pooling of values, it is much faster to extract than SIFT-like descriptors [25]. Based upon BRIEF, Rublee proposed the Oriented FAST and Rotated BRIEF (ORB) matching algorithm in ICCV 2011 [26,27]. ORB algorithm can achieve similar effects to SIFT algorithm, and its speed can be about 100 times faster than that of SIFT algorithm. In feature point detection and description process, the ORB algorithm uses the Features from Accelerated Segment Test (FAST) algorithm [28] and the Binary Robust Independent Elementary Features (BRIEF) algorithm [23,29]. ORB algorithm combines the real-time advantages of the above two algorithms to improve the computational efficiency. Unlike random sampling or unsupervised learning of pairs, the Binary Robust Invariant Scalable Keypoints (BRISK) [20] uses hand-crafted, concentric ring-based sampling modes. BRISK uses pixel pairs with large distances between them to calculate the orientation of the patch centered the feature point, and pixel pairs separated by the short distance to calculate the value of the descriptor itself again by performing a binary intensity comparison on pixel pairs. Inspired by the retinal pattern of human eyes, the Fast Retina Keypoint descriptor (FREAK) was proposed in [18]. Similar to BRISK, FREAK also uses concentric rings, but unlike BRISK, FREAK samples more points exponentially in the inner ring. Of all the possible pixel pairs may be sampled, FREAK uses unsupervised learning to choose an optimal set of point pairs. Similar to BRIEF, the Local Difference Binary (LDB) descriptor was proposed in [30,31]. Instead of comparing smoothed intensities, mean intensities comparisons of grids with size was used in LDB.

In addition to the mean intensity values, LDB compares the mean values of horizontal and vertical derivatives, amounting to 3 bits per comparison. Inspired by the local binary descriptor, a novel superpixel region binary description (SRBD) method is proposed in [32]. SRBD is a dominant orientation difference vector constructed based on the differences between the current superpixel and its neighborhoods. In 2017, a novel approach was proposed to generate a binary descriptor optimized for each image patch independently, which combines the advantages of efficient binary descriptors with the improved performance of learning-based descriptors [33]. The approach is inspired by the linear discriminant embedding that simultaneously increases inter-class distances and decreases intra-class distances. A set of discriminative and uncorrelated binary tests is established from all possible tests in an offline training process. The patch adapted descriptors are then efficiently built online from a subset of features which lead to lower intra-class distances and thus, to a more robust descriptor. In 2019, Ma Dan and Lai Huicheng proposed an algorithm for remote sensing image matching based on the ORB algorithm and the NSCT algorithm, which can make up for the lack of scale invariance of ORB algorithm and has more robust and comprehensive consideration in the complex situation [34]. The authors of [35] carried out researches on randomized local binary features under the background of keypoints recognition and image patches classification. They conclude that the quality of the binary feature space can be greatly improved by increasing the randomness of the basic sampling operator. In addition to image matching methods based on traditional manual labeling, current image processing methods based on deep learning are also widely used to solve image matching problems. Numerous deep learning networks have also been applied to extract image features. To address the problem of cross-modal information retrieval in the domain of remote, a novel deep neural network-based architecture which is considered to learn a discriminative shared feature space for all the input modalities was proposed in [36]. A new large-scale remote sensing image retrieval method based on deep hash neural network (DHNN), was proposed which can automatically learn good feature extraction operations and feature hash maps under the supervision of labeled samples [37]. To cope with CS-LSRSIR, a source-invariant deep hashing convolutional neural networks (SIDHCNNs) was proposed in [38], which can be optimized in an end-to-end manner using a series of well-designed optimization constraints. A novel DML method named as global optimal structured loss to deal with the limitation of triplet loss [39]. To improve the problem that the pair-based loss currently used in DML is not appropriate, a novel distribution consistency loss was proposed in [40]. 

Deep learning-based methods can extract subtle underlying features and abstract high-level features and can more accurately describe features. However, some deep learning networks have complex structures and are computationally expensive. The traditional feature description algorithm based on manual annotation, especially the algorithm based on binary feature description, has certain advantages in terms of calculation speed and calculation cost.

The runtime advantages make binary description more suitable for real-time applications and handheld devices. However, binary description has lower recognition capabilities because of the oversimplified information such as a raw intensity of a subset of pixels in an image patch are used for binary testing. Our work was proposed based upon ORB algorithm. When ORB is used to match with large databases, the lack of uniqueness leads to a large number of mismatches. According to the principle of ORB, it has the following shortcomings:

1. In the feature description process, the ORB algorithm uses the BRIEF feature description method with direction information. By analyzing the principle of the BRIEF algorithm, it can be found that the BRIEF algorithm usually compares the gray size relationships of pixel pairs directly to determine the corresponding coding value (0 or 1) of the binary coding. The gray value of a single pixel is so sensitive to noise that a small change in image contents can cause a large change in the gray value, making the BRIEF algorithm very sensitive to noise. In order to reduce the impact of noise, the ORB algorithm firstly filters the image and uses pixel patch pairs comparison method. Although the filtering process can reduce the sensitivity to noise to a certain extent, it may also cause the loss of high-frequency region information of images.

2. The BRIEF algorithm calculates the feature description based on the comparison of the pixel pairs. In the comparison process, only the gray size relationship of pixel pairs is considered, while the detailed gray difference value information between the pixel pairs is ignored, which may cause the loss of partial image information. Obviously, this partial image information is also useful for improving the discriminability of the description operator [41]. This leads to the description operator being incomplete. If the size relationship and the difference value information above can be utilized together, the relationship between the neighborhood pixels can be completely expressed, thereby improving the discriminating ability of the descriptor.

In this paper, we focus on the feature description process and propose an improved ORB algorithm, which can further improve the performance of the original ORB algorithm. Compared with original ORB algorithm, the following two improvements have been made:

Step 1: Instead of pixel patch pairs comparison used in original ORB algorithm, a three-pixel patch groups comparison method is used in our paper. In each group, the gray value of the main patch is compared with that of the other two companion patches to generate the binary string. By this method, visual information with more spatial support is considered for each bit of the descriptor, making their final values therefore less sensitive to noise.

Step 2: The gray difference value of the three-pixel patch groups comparison in Step 1 can be recorded. These gray difference values can be converted to another binary string by a specified threshold. In our method, the feature fusion method is adopted to generate a new description by connecting the above two binary strings.

## 2. Related Algorithm

### 2.1. ORB Algorithm and BRIEF Algorithm

As a feature point detection and description algorithm based on visual information, ORB algorithm combines FAST corner detection and BRIEF feature description. In feature description stage, ORB uses BRIEF feature description method to describe the feature point by improving its primary drawback that without rotational invariance and having sensitivity to noise. 

The main idea of the BRIEF is to randomly select pixel points pairs around the feature point. Then the comparison of the gray value of the selected pixel points pairs is used to produce a binary string as the feature description. Specifically, define a feature point P and select a neighborhood W centered on the point P. In the neighborhood W, n pairs of pixel points are selected randomly with points coordinates obeying the Gaussian distribution. A binary test method τ in Equation (1) is defined to calculate each bit of the final binary string. For the uth pixel points pair of the above n pixel points pair, there are two points $p_{u1}=\left(x_{u1},y_{u1}\right)\;$ and $p_{u2}=\left(x_{u2},y_{u2}\right)$. The gray values of $p_{u1}$ and $p_{u2}$ are compared by the following Equation (1)

(1) $$\tau \left(f;\left(p_{u1},p_{u2}\right)\right)=\{\begin{array}{c} 1,atiof\left(p_{u1}\right)<f\left(p_{u2}\right)\\ 0,atiof\left(p_{u1}\right)\geq f\left(p_{u2}\right) \end{array}$$

where $f\left(p_{u1}\right)$, $f\left(p_{u2}\right)$ are the gray values of random points $p_{u1}$ and $p_{u2}$ respectively. For feature point P, its feature description can be formed as a vector of n binary tests based on n pixel points pairs as

(2) $$F_{n}\left(P\right)=\underset{1\leq u\leq n}{\sum }2^{u-1}\tau \left(f;\left(p_{u1},p_{u2}\right)\right)$$

where n can be 128, 256, and 512, occupying 16 bytes, 32 bytes, and 64 bytes respectively. Considering the generation speed, the distribution and accuracy of descriptors, a 256-dimensional descriptor is used by the ORB algorithm.

The BRIEF algorithm generates feature description by comparing the gray values of the two points in the selected pairs in the neighbor window of the feature point. This method is very sensitive to noise. To eliminate the effects of noise, the ORB algorithm has made some improvements. An image window area of  31×31 pixels size around the feature point is defined, a certain number of image patches of 5×5 pixels size is randomly selected, and the binary string is generated using the gray integration of these image patches. In addition, the advantage of the BRIEF algorithm is its high speed, but it does not have rotational invariance. The ORB algorithm solves this problem by measuring the directions of the feature point using the gray centroid method. To attain a description of n bits, n matching pairs of pixel points need to be selected. For example, a 2×n matrix Q can be defined as

(3) $$Q=\left[\begin{array}{c} x_{1},x_{2},\cdots \cdots ,x_{n}\\ y_{1},y_{2},\cdots \cdots ,y_{n} \end{array}\right]$$

After obtaining the rotation direction θ of the feature point, the corresponding rotation matrix $R_{\theta }$ can be obtained, and then a rotation matching pair matrix $Q_{\theta }=R_{\theta }Q$ can be constructed. With the same $F_{n}(p)$ in Equation (2), a descriptor with rotation invariance can be obtained as

(4) $$F_{n}\left(P,\theta \right)=F_{n}\left(P\right)\;|\;\left(x,y\right)\in Q_{\theta }$$

### 2.2. Local Binary Patterns

Our work is related to a particular variant of the Local Binary Patterns (LBP), so we make a brief introduction for LBP. LBP, proposed as global (whole image) representation by [42,43,44], is an effective method to describe the texture feature, which has been successfully applied to many image classification problems, most notably of texture and face images. LBP produces for each pixel in the image a binary string representation. In fact, to our knowledge, 8-bit strings or less were employed in all applications of LBP. These bits, similarly to the binary descriptors, are set following binary comparisons between image pixel gray intensities. In the original LBP implementation, these bits were computed by using a pixel’s value as a threshold, applied to its eight immediate spatial neighbors, and taking the resulting zero/one values as the 8-bit string. By using only 8-bits, each pixel is thus represented by a code in the range of 0 to 255 (or less, in some LBP variations), which are then pooled spatially in a histogram in order to represent image portions or entire images [45].

Our work is related to a particular variant of LBP, named three-patch LBP (TPLBP), which is an exceptionally potent global representation for face image [44]. TPLBP codes are produced by comparing the values of three patches to produce a single bit value in the code assigned to each pixel. In [44], another particular variant of LBP, named four-patch LBP (FPLBP) was also proposed. FPLBP codes are produced by comparing the values of four patches to produce a single bit value in the code assigned to each pixel. The authors of [44] used LBP, TPLBP and FPLBP to test the same data set. Experimental result is shown in Table 1. Table 1 shows that TPLBP can achieve higher recognition ability than LBP and FPLBP.

Although the algorithm proposed in [44] is used for face recognition, it cannot be directly applied to image matching. However, its essence is also the feature description generated by comparing pixel patches. Therefore, in our paper, we adopt a feature description generation method based on three-pixel patch comparison.

## 3. Proposed Feature Descriptor

### 3.1. Three-Patch Method

Compared with other matching algorithms, although BRIEF has the advantages of fast speed, it cannot solve the problem of noise sensitivity very well and does not have rotation invariance. ORB uses mean filtering to reduce noise sensitivity and calculates the principal direction of feature points to solve the problem of rotation invariance. Regardless of the fact that mean filtering can alleviate some problems, it will lead to information loss, especially in high-frequency areas where key points are often detected. To improve this shortcoming, a three-pixel patch groups comparison method is adopted in our paper. In each three-pixel patch group, the main patch is compared with the other two companion patches respectively to generate the binary description. By this way, visual information with more spatial support is considered for each bit of the descriptor, and therefore the descriptor is less sensitive to noise [45].

**Figure 1:**
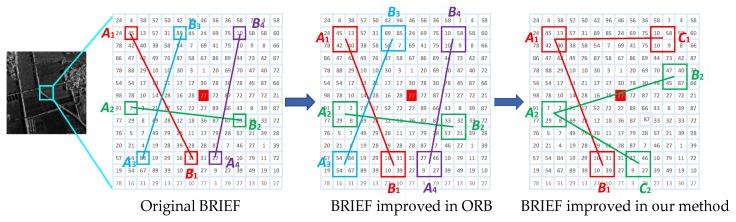
Gray value comparison modes of the BRIEF, ORB, and improved ORB.

The principle of the three-patch method is shown in Figure 1. The BRIEF algorithm calculates the feature descriptor as a binary string. It is to select n pairs of pixel points $(A_{i},B_{i})\left(i=1,2,\ldots ,n\right)$ in the neighborhood of each feature point. The magnitude of the gray value of each point pair is then compared. If the gray value of $A_{i}$ is higher than that of $B_{i}$, the corresponding bit in the binary string is set to 1, otherwise it is set to 0. All point pairs are compared to produce a binary string of length n. Generally, n is 128, 256, or 512, and usually 256. In order to reduce the sensitivity to noise, the ORB algorithm improves the comparison of pixel pairs in the BRIEF algorithm to the comparison of pixel patches pairs. This method improves the anti-noise performance of the algorithm to some extent. 

We refer to the method of [44] and improve the comparison of pixel patch pairs to three-pixel patch pairs based on the ORB algorithm. This method can obtain more image information and get more detailed descriptions of feature points. First, randomly select n three-pixel patch groups in the neighborhood window of the feature point, which is different from the eight-pixel patches of three-patch LBP (TPLBP) in [44]. In order to briefly describe the principle of the algorithm, in Figure 1, we only show two sets of three-pixel patch groups. In each group of three-pixel patches, one pixel patch $A_{i}$ is determined as the main pixel patch, and the other two pixel patches $B_{i}$, $C_{i}$ are companion pixel patches. Then calculate the magnitude relationship between the gray value of the main pixel patch and the two companion pixel patches. Only when the gray value of the two companion pixel patches $B_{i}$, $C_{i}$ is smaller than the main pixel patch $A_{i}$, the corresponding position of the feature description vector is 1, otherwise it is 0.

The specific steps of the algorithm proposed in this paper are as follows. Specifically, define a feature point P, and select a neighborhood window W of M× M size centered the feature point P. In the neighborhood window *W*, T three-patch groups are selected according to the Gaussian distribution, with each group including three-pixel patches. The coordinates of the center point of each pixel patch are

(5) $$Z={\left\{z_{t}\right\}}_{t=1\ldots T}={\left\{\left[\left(x_{t1},y_{t1}\right),\left(x_{t2},y_{t2}\right),\left(x_{t3},y_{t3}\right)\right]\right\}}_{t=1\ldots T}$$

According to the three-patch method principle, for each three-patch group, three-pixel patches of k×k(k<M) size respectively centered on point coordinates $\left(x_{t1},y_{t1}\right)$, $\;\left(x_{t2},y_{t2}\right)$, $\left(x_{t3},y_{t3}\right)$ defined in Equation (5) can be obtained as

(6) $$S={\left\{S_{t}\right\}}_{t=1\ldots T}={\left\{\left[A_{t},B_{t},C_{t}\right]\right\}}_{t=1\ldots T}\;$$

where patch $S_{t1}$ is defined as the main patch, and $S_{t2}$, $S_{t3}$ are defined as the companion patches of $S_{t1}$.

The value of the corresponding bit of the binary string is determined as

(7) $$g\left(W,S_{t}\right)=\{\begin{array}{l} 1,\;f\left(A_{t}\right)<f\left(B_{t}\right)\;\&\;f\left(A_{t}\right)<f\left(C_{t}\right)\\ 0,\;\mathrm{otherwise} \end{array}$$

The number of three-patch groups can be 128, 256, or 512. The length of the binary string is correspondingly be 128, 256, or 512 bits. According to Equation (7), a binary string $b_{W}$ is formed by comparing all T three-patch groups in window W, defined as

(8) $$b_{W}=\underset{1\leq u\leq n}{\sum }2^{t}g\left(W,S_{t}\right)$$

### 3.2. Binary String Using Gray Difference Value Information

**Figure 2:**
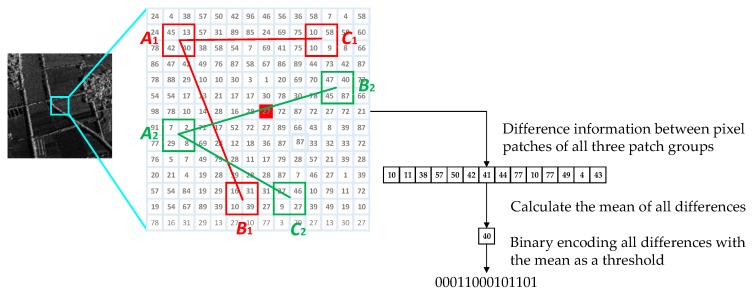
Binary string using gray difference value information.

The binary string described in Section 3.1 is formed by comparing the gray values of pixel patches in each group. However, gray difference information is ignored, resulting in a partial loss of image information. If the gray values and gray difference value information of pixel patches in each group can be utilized together, the relationship of the neighborhood pixel points can be completely expressed, thereby improving the discriminating ability of the description. In this paper, we propose a feature description method combining gray magnitude relationship and gray difference value information. Equation (8) in Section 3.1 describes a binary coding method in which three patches in a group are compared by our specified way to generate a binary string. Equation (8) records the gray magnitude relationships between the pixel patches, but it does not calculate gray difference value information. As shown in Figure 2, in order to make full use of the difference information between pixel patches, we first calculate the difference between the main pixel patch and the companion pixel patches in all three-pixel patch groups. Then calculate the average of all the differences as a threshold to perform binary quantization of all the differences. For each three-pixel patch group, only when the difference between the main pixel patch and the other two companion pixel patches are greater than the threshold, the corresponding bit of the binary encoding is set to 1, otherwise it is set to 0. After all the three-pixel patch groups are calculated, we can get a binary coded string as a feature description based on local gray difference information.

The detailed steps are as follows. Gray difference values Q can be defined as

(9) $$Q=\;{\left\{Q_{t}\right\}}_{t=1\ldots T}={\left\{Q_{t1}=\left|f\left(A_{t}\right)-f\left(B_{t}\right)\right|,Q_{t2}=\left|f\left(A_{t}\right)-f\left(C_{t}\right)\right|\right\}}_{t=1\ldots T}$$

For each three-patch group, two gray difference values can be recorded. 2×T difference values can be generated for a feature point. Since floating-point gray difference values cannot be directly encoded as 0 or 1, the mean value of all the difference values is calculated as the threshold $Q_{average}$ as

(10) $$Q_{average}=\frac{1}{T}\underset{1\leq t\leq T}{\sum }Q_{t}=\frac{1}{2T}\underset{1\leq t\leq T}{\sum }\left(Q_{t1}+Q_{t2}\right)$$

In Equation (10), two gray difference values are generated. The average value of these two gray difference values is used to determine the value (for example 0 or 1) of the corresponding bit of the binary feature description as

(11) $$h\left(W,Q_{t}\right)=\{\begin{array}{l} 1,\;Q_{average}<Q_{t1}\;\&\;Q_{average}<Q_{t2}\\ 0,\;\mathrm{otherwise} \end{array}$$

According to Equation (11), the binary string bw^ based on gray difference values can be obtained as

(12) bw^=∑1≤t≤T2th(W,Qt)

The length of bw^ depends on the number of the selected three-patch groups defined as T in Equation (8). In Section 3.1, T can be 128, 256, or 512. In this paper, T is set to 128.

### 3.3. Feature Description Fusion

Connecting $b_{W}$ generated in Section 3.1 and bw^ generated in Section 3.2, the new binary description of the feature point is obtained as

(13) Descriptionkeypoint=bW+bw^

**Figure 3:**
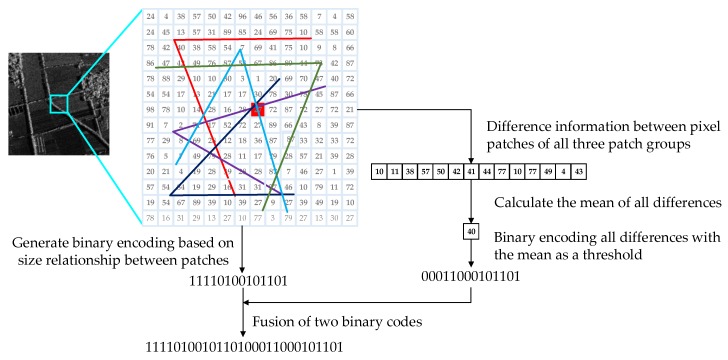
Feature fusion process proposed in this paper.

Feature fusion process proposed in this paper is shown in Figure 3. We proposed a feature description method combining gray magnitude relationship and gray difference value information. The length of the new description is twice as long as that of the description generated by the ORB algorithm. In this paper, the length of the new description is set to 512 bits. Although the length of the new description is twice as long as that of the ORB, the distance between the binary strings can be calculated using the Hamming distance, which can be quickly implemented using an exclusive XOR operation.

### 3.4. Proposed Algorithm Process Overview

**Figure 4:**
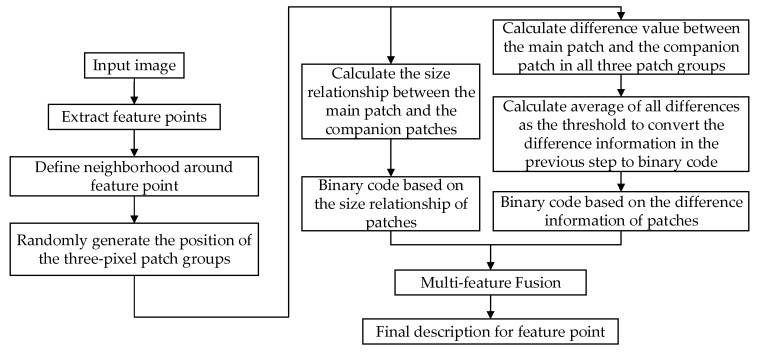
Final description process proposed in this paper.

As shown in Figure 4, we first complete the feature point extraction, define the neighborhood image window with the feature point as the center, and then randomly select the location of the three-pixel block in the neighborhood. Then complete binary coding based on pixel block size relationship and binary coding based on pixel block gray level difference information in the neighborhood, and finally combine the two binary codes through feature fusion to generate a new feature description as the definitive description of feature points. 

The steps of the feature description algorithm proposed in this paper are as follows: in this paper, M is set to 48 pixels, T is set to 128,  k is set to 7 pixels, f() is the gray mean function.
**Algorithm for ORB-TPLGD****Input: **Image data**Output:** Feature description vector for each feature point1: Extract feature points set $P=\left\{p_{1},p_{2},\ldots ,p_{i},\ldots ,p_{N}\right\}$ from input image2:** for**
i=1,2,…,N
**do**3:   Define neighborhood W of M× M around feature point $p_{i}$ 4:    In W, T three-patch groups are selected using Gaussian function, coordinates of the center point is $Z={\left\{z_{t}\right\}}_{t=1\ldots T}={\left\{\left[\left(x_{t1},y_{t1}\right),\left(x_{t2},y_{t2}\right),\left(x_{t3},y_{t3}\right)\right]\right\}}_{t=1\ldots T}$
5:   Define k×k size patches $S_{t1},S_{t2},S_{t3}$ centered on point $\left(x_{t1},y_{t1}\right),\left(x_{t2},y_{t2}\right),\left(x_{t3},y_{t3}\right)$, $S={\left\{S_{t}\right\}}_{t=1\ldots T}={\left\{\left[S_{t1},S_{t2},S_{t3}\right]\right\}}_{t=1\ldots T}$
6:   **for**
j = 1,2,…,T
**do** 7:      **if **$f\left(S_{t1}\right)<f\left(S_{t2}\right)\;\&\;f\left(S_{t1}\right)<f\left(S_{t3}\right)$ then bit = 1 8:      **else** then bit = 09:      **end if**10:   **end for** (obtain a binary string $b_{W}$) 11:   Calculate the difference within all three pixel patches groups $Q=\;{\left\{Q_{t}\right\}}_{t=1\ldots T}={\left\{Q_{t1}=\left|f\left(S_{t1}\right)-f\left(S_{t2}\right)\right|,Q_{t2}=\left|f\left(S_{t1}\right)-f\left(S_{t3}\right)\right|\right\}}_{t=1\ldots T}$
      Calculate the mean of all differences $Q_{average}$
12:   ** for**
j = 1,2,…,T
**do**13:      **if **$Q_{average}<Q_{t1}\;\&\;Q_{average}<Q_{t2}$ then bit = 1 14:      **else** then bit = 0 15:      **end if**16:   **end for** (obtain a binary string bw^) 17:     $b_{W}$ and bw^ are fused to get final $Description_{keypoint}$18: **end for**


### 3.5. Three-Patch Group Arrangements

**Figure 5:**
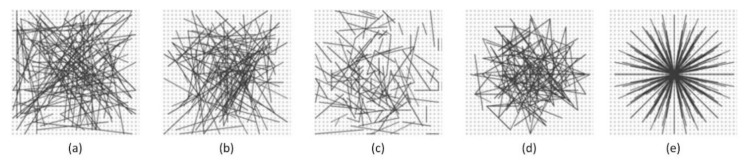
Different approaches to choosing the test locations. (**a**) (X,Y) conforms to Uniform $\left(-\frac{S}{2},\frac{S}{2}\right)$, (**b**) (X,Y) conforms to Gaussian $\left(0,\frac{1}{25}S^{2}\right)$, (**c**) X conforms to Gaussian$\left(0,\frac{1}{25}S^{2}\right)$ and Y conforms to Gaussian $\left(x_{i},\frac{1}{100}S^{2}\right)$, (**d**) The $\left(x_{i},y_{i}\right)$ are randomly sampled from discrete locations of a coarse polar grid introducing a spatial quantization, (**e**) $x_{i}={\left(0,0\right)}^{T}$ and $y_{i}$ takes all possible values on a coarse polar grid containing N pixel point pairs [28].

Our method is proposed based on ORB algorithm. The ORB algorithm adopts the improved BRIEF algorithm in the feature description stage. In [28], the authors tested five different sampling approaches to randomly select pixel points pairs as illustrated in Figure 5. Generating a length N bits vector leaves many options for selecting N test locations $\left(x_{i},y_{i}\right)$ in a neighborhood window of size S×S centered the feature point.

For each of these sampling approaches, [28] computes the recognition rate. The symmetrical and regular Figure 5e strategy loses out against all random designs Figure 5a–e, with Figure 5c enjoying a small advantage over the other three in most cases. For this reason, Figure 5b sampling method is employed in all further experiments in [28].

Based on the above conclusions, we also adopt Figure 5b sampling method to select random three-patch groups in our paper. Even small detection windows give rise to a huge number of possible three-patch group arrangements. Considering that only a small number of arrangements are typically required, we must therefore consider which of the many possible three-patch group arrangements should be employed. We first set up a training set based on Middlebury stereo dataset. Middlebury is a stereo dataset, with each part published in five different works in the years 2001, 2003, 2005, 2006, 2014, respectively in [46,47,48,49,50]. The image pairs of this database are indoor scenes taken controlled lighting conditions, and the density and precision of true disparities are high via using structured light measurement. The dataset is divided into 35 training sets, and the resolution of image pairs is selected the smallest size of the given configuration. We set up a training set of 20000 points pairs, separately drawn from corresponding images pairs in the Middlebury dataset.

We form 10000 three-patch groups arrangements. For each arrangement, T three-patch groups are defined by selecting the center pixel coordinates $\left(x_{t1},y_{t1}\right)$ of the main patch $S_{t1}$ and $\left(x_{t2},y_{t2}\right)$, $\left(x_{t3},y_{t3}\right)$ of the two companion patches $S_{t2}$ and $S_{t3}$ in each three-patch group. The selection of these pixel points coordinates obeys the Gaussian distribution equation: (X,Y) conforms to Gaussian $\left(0,\frac{1}{25}S^{2}\right)$, where is the size of the neighborhood window. In this paper, the size of neighborhood window is set to 48×48 referring to [45], The pixel patch size is set to 7×7, and the choice reason is described in the Section 4.4. We then evaluate each of these 10k arrangements over all the 20000 points pairs in training set to find the best performed arrangement. We define the quality of an arrangement by summing the number of times it correctly yielded the same binary value for the two-pixel points in a point pair among the above training set of 20000 points pairs.

## 4. Experiments and Result

The experimental equipment is 64-bit Win10 platform (Intel Core i7-7700 CPU, primary frequency 3.60GHz, 16GB memory). The experiment is carried out with Microsoft visual studio 2017 combined with opencv3.4.0. The Oxford dataset and a series of SAR images dataset are selected as the test image datasets. Our improved ORB algorithm is compared with the state-of-the-art algorithms SIFT, SURF, BRIEF, BRISK, FREAK, LDB, and ORB.

According to the image matching process, the first step is to detect feature points. In the feature point detection process, SIFT, SURF, BRISK, and FREAK algorithms adopt their respective detection methods. BRIEF, LDB, ORB, and our improved ORB all adopt FAST corner detection method used in ORB algorithm. In the feature point matching process, SIFT and SURF algorithms use the Euclidean distance to match because of their feature vectors are floating-point data. The BRIEF, BRISK, FREAK, LDB, ORB, and our improved ORB use Hamming distance to compute similarity. Finally, RANSAC algorithm is used for all tested algorithms to eliminate mismatches. A keypoint pair is considered to be a correct match if the error of true match is within 3 pixels. Detailed configuration information is shown in the Table 2.

### 4.1. Evaluation Metrics

After the matching is completed, we can obtain a matching feature points pairs set, which contains correctly matched point pairs and incorrectly matched point pairs. The matching precision rate refers to the number of correctly matched point pairs among all matched point pairs in the two images. According to the literature [51], we first need to know the number of matching feature points in one-to-one correspondence in the two images. For the two matching images A and B, there may be a change in vision, or a rotation or scale change. Therefore, it is assumed that the homography matrix for the mapping from image A to image B is $A_{H}$, and the homography matrix for image B to image A is $B_{H}$. Suppose $N_{1}$ feature points are detected in image A and $N_{2}$. feature points are detected in image B. According to the homography matrices $A_{H}$ and $B_{H}$, the coordinates of the image A in the image B and the coordinates of the B image in the A image are obtained, the unqualified feature points are excluded, and the common feature points $n_{1}$ and $n_{2}$ in the two images are obtained. Take $n=\min\left(n_{1},n_{2}\right)$ as the common feature points of the two images. Then get the distance $dist\left(H_{A}*n_{A},\;n_{B}\right)$ according to the feature point n. If $dist\left(H_{A}*n_{A},\;n_{B}\right)$ is less than the given threshold ε, then these feature points are considered to be the common feature points of the two images, and there is a one-to-one correspondence relationship, thereby obtaining the number of feature point matches in the two images. This number is the number of point pairs that should be matched in the current two images.

We define the number of matches obtained by the image matching algorithm as $Num_{all\;matches}$, and the number of actual matching between images is $Num_{true\;matches}$. Through the one-to-one corresponding feature point coordinate true values obtained above, we can calculate the distance between the position of the matching point and the true value obtained by the image matching algorithm. When this distance is greater than a certain threshold, we consider this a wrong match. In this paper, we set this threshold to 3 pixels. After traversing all the matching point pairs, we can get all the correct matches, which is defined as $Num_{correct\;matches}$.

In experiment, four evaluation criterions are used to measure the performance of the algorithms. The first criterion is precision [51], based on the number of correct matches ($Num_{correct\;matches}$) with respect to the number of all matched points ($Num_{all\;matches}$). The equation is

(14) $$\mathrm{Precision}=\;\frac{Num_{correct\;matches}}{Num_{all\;matches}}\;$$

The second criterion is recall [51], based on the number of correct matches ($Num_{correct\;matches}$) with respect to the number of corresponding points ($Num_{true\;matches}$) between input image pairs. It can be expressed as

(15) $$\mathrm{Recall}=\;\frac{Num_{correct\;matches}}{Num_{true\;matches}}\;$$

The third criterion is root-mean-square error (RMSE). Calculate the root mean square error based on the position of the detected matching point and the position of the real matching point. The root-mean-square error can intuitively show the difference between the position of the detected matching point and the real position. Suppose a pair of matching points $\;p\left(x_{p},y_{p}\right)$, $q\left(x_{q},y_{q}\right)$. According to the true value, the true position of p point on the other image which includes q is $\tilde{p}\left(\tilde{x_{p}},\tilde{y_{p}}\right)$. The calculation equation of RMSE is

(16) $$\mathrm{RMSE}=\;\sqrt{{\left(x_{q}-\tilde{x_{p}}\right)}^{2}+{\left(y_{q}-\tilde{y_{p}}\right)}^{2}}\;$$

The fourth criterion is image matching time. In this paper, matching time is defined as the time spent from the beginning of feature point extraction to the end of feature point matching. Images reading and the subsequent calculation of performance indicators of algorithms are not included.

### 4.2. Oxford Dataset

**Figure 6:**
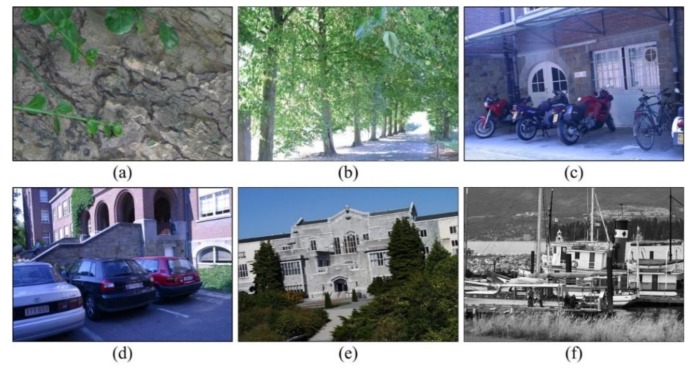
Base images provided by Oxford dataset. (**a**) Wall (viewpoint), (**b**) trees (blur), (**c**) bikes (blur), (**d**) Leuven (illumination), (**e**) Ubc (JPG compression), (**f**) boat (rotation and scale).

Oxford dataset is a publicly measurable database provided in [51]. We select six image groups from the Oxford dataset as shown in Figure 6. Each image group represents different image change relationships including viewpoint changes (Wall group, Graf group), image blur (tree group, bike group), illumination (Leuven group), JPG compression (Ubc group), rotation scale conversion (bark group, boat group).

Each group includes six images, the first image is the reference image, and the second to the sixth images are the images used to be matched. The first image can be matched with the other five images as an input image pair. Images in PPM format and corresponding homography matrix for each image pair are also provided by the Oxford dataset. Based on the homography matrix, the matching precision and recall can be calculated. For each image group, the first image and the remaining five images are matched separately.

### 4.3. SAR Dataset

**Figure 7:**
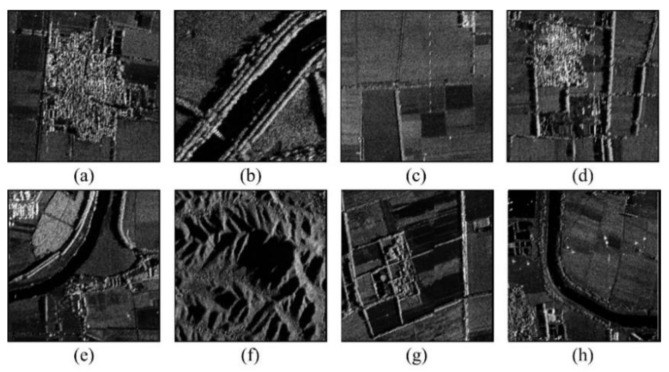
Eight selected SAR Images of Bedfordshire. (**a**) Village, (**b**) river, (**c**) field, (**d**) village, (**e**) river, (**f**) hill, (**g**) village, (**h**) river.

In this section, a series of SAR images, shown in Figure 7, obtained by the unmanned airborne SAR platform, are used to test the performance of our improved ORB algorithm. These SAR images are X-wave band SAR images of Bedfordshire in the southeast of England, with 3-meter resolution. Taking a SAR image as the reference image, it can be rotated by a certain angle and scaled at a certain scale to obtain a transformed image. Subsequent matching uses this SAR image and its transformed image as a test image pairs. We can calculate the new coordinates of the transformed image points based on given angle and scale, and thus the ground-truths of the image pair can be determined.

### 4.4. Descriptor Size and Patch Size Test

In the tests reported above, we used a descriptor of 64 bytes. Here, we revisit the tests on the Oxford dataset in order to evaluate the effect descriptor size has on its performance. We test varying descriptor sizes using 4, 8, 16, 32, 64, and 128 bytes for the representation.

One of the key components of our new descriptor is the use of pixel patches compared to sampling single pixels. Another parameter that affects the descriptor is the patch size in each three pixels patches group. We next evaluate the effect of pixel patches size on the performance of our new descriptor. Here, we use a 64-byte descriptor representation, testing it with a patch sizes ranging from 5×5 to 17×17.

Table 3 summarizes descriptor sizes test results. When testing certain descriptor sizes and patch sizes, the output is abnormal when matching the last image of some image groups. Therefore, this test only uses the first five images in each Oxford image group for testing.

The above data shows that, in general, the longer the descriptor length, the better the result. However, as the length of the descriptor increases, the effect that can be improved also becomes smaller. In terms of the patch size, in nearly all cases, the bigger the patches used, the higher the performance gain. In this paper, considering the promotion effect and efficiency, the descriptor size is set to 64 bytes, and the patch size is set to 7×7.

### 4.5. Experiment Result Based on Oxford Dataset

#### 4.5.1. Matching Result Based on Oxford Dataset

**Figure 8:**
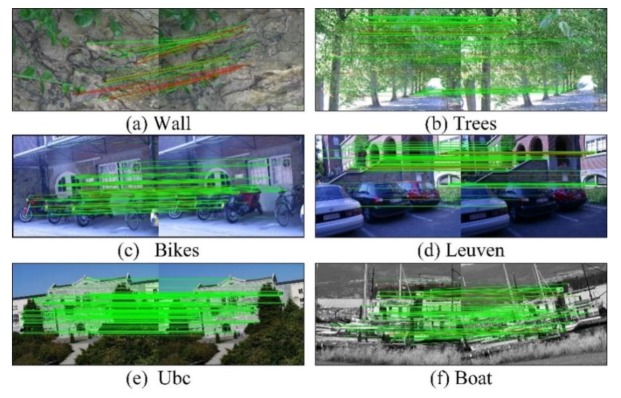
Matching effect of images from Oxford dataset using our method. (**a**) Wall (viewpoint), (**b**) trees (blur), (**c**) bikes (blur), (**d**) Leuven (illumination), (**e**) Ubc (JPG compression), (**f**) boat (rotation and scale).

After image matching completed, matching effect of our improved ORB algorithm is shown in Figure 8, in which the correct matched points pairs are connected by the green line while red lines referring to the wrong matched points pairs. Meanwhile, the matching precision, recall, RMSE and operation time of each group of images are recorded. The symbol ORB-TPLGD represents the improved ORB algorithm we proposed in this paper.

The first image of each image group is the reference image, and the other five images are used to matched. The changes from the second image to the sixth image become larger. The matching precision of the latter image is generally less than the previous image.

#### 4.5.2. Matching Precision and Recall Based on Oxford Dataset

**Figure 9:**
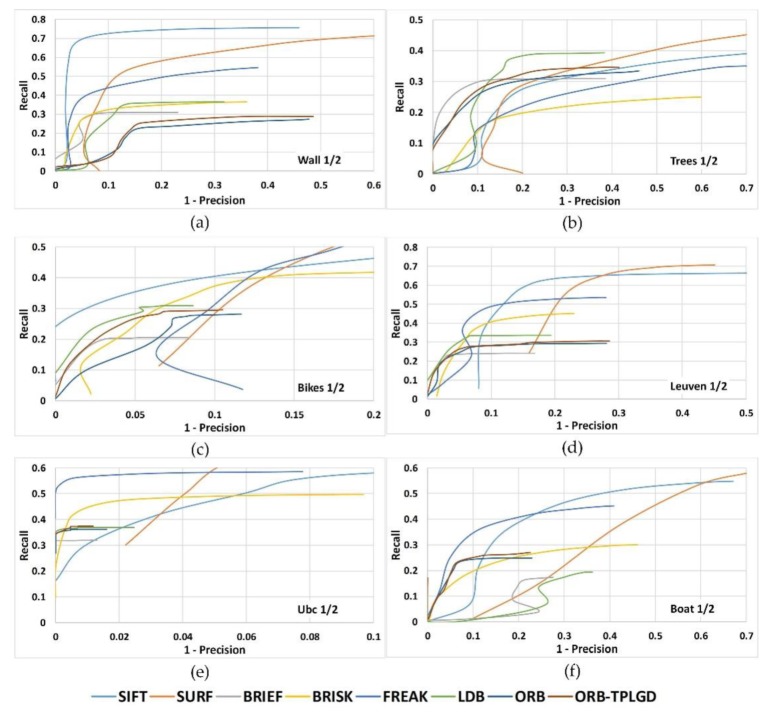
Recall versus 1-precision curves for image pairs 1/2 of all six image sequences. (**a**) Wall (viewpoint), (**b**) Trees (blur), (**c**) Bikes (blur), (**d**) Leuven (illumination), (**e**) Ubc (JPG compression), (**f**) Boat (rotation and scale).

To examine the distinctiveness of binary descriptors we plot the Recall versus 1-precision curves. The threshold value of the distance ratio is varied to obtain the curve of the average recall versus average 1-precision. Figure 9 shows the recall versus 1-precision curves for image pairs 1/2 of all six image sequences. Results show that ORB-TPLGD proposed in this paper outperforms the original ORB descriptor for all six image sequences.

Table 4 records the average matching precision of these eight image matching algorithms based on six groups of Oxford dataset respectively. According to Table 4, our algorithm can achieve better matching performance in matching precision, respectively with 12.746%, 5.135%, 4.350%, 7.062%, and 1.159% higher than SURF, BRIEF, FREAK, LDB, and ORB.

The average matching recall is counted in Table 5. From Table 5, as for those algorithms using binary feature, the recall of our improved ORB algorithm is higher than that of BRIEF and ORB by 0.998% and 1.078% and lower than that of BRISK, FREAK and LDB by 4.503%, 14.594%, and 2.628%. In this paper, the matching algorithms using binary feature description all adopt the same feature point detection method. Therefore, recalls in this experiment are more dependent on the feature description method and matching method.

#### 4.5.3. Matching RMSE Based on Oxford Dataset

**Figure 10:**
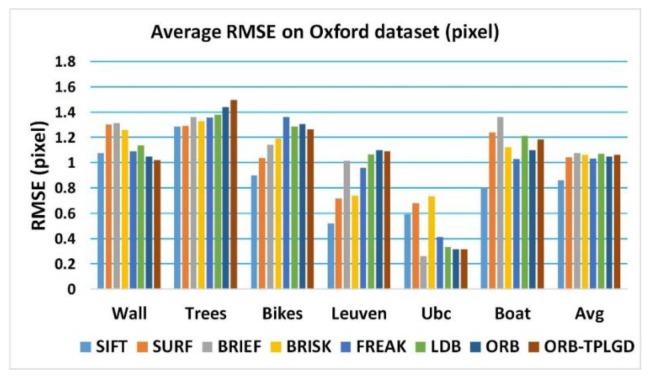
Average matching RMSE based on Oxford dataset.

Figure 10 shows the average matching RMSE of these eight tested image matching algorithms based on six groups of Oxford dataset respectively. In most cases, our improved ORB algorithm can achieve a similar level of RMSE as the current related algorithms. The average matching RMSE is counted in Table 6. Results show that the positioning accuracy of SIFT algorithm is the highest, and the positioning accuracy of algorithms based on binary feature description is generally lower than SIFT algorithm.

#### 4.5.4. Matching Time Based on Oxford Dataset

**Figure 11:**
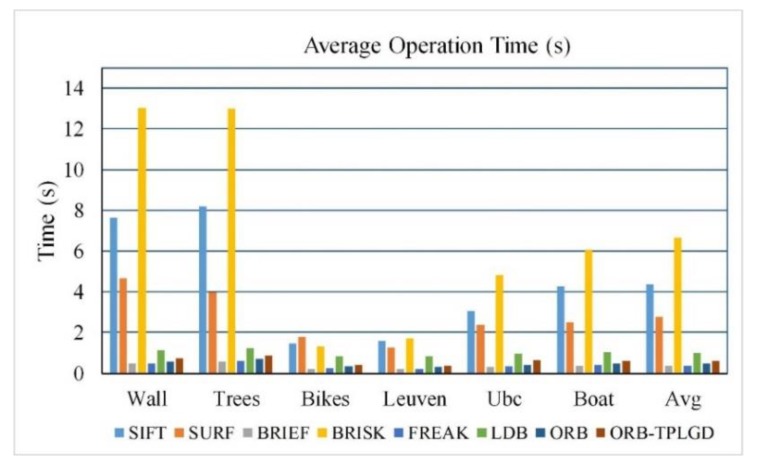
Average matching time based on Oxford dataset.

Table 7 calculates the average matching time of the eight algorithms tested. As shown in Table 6 and Figure 11, in terms of operational efficiency, the time SIFT and SURF algorithm required is much longer than that of BRIEF, FREAK, LDB, ORB, and ORB-TPLGD algorithms. BRIEF is the most efficient followed by FREAK, ORB, and our improved ORB-TPLGD.

### 4.6. Experiment Result Based on SAR Dataset

#### 4.6.1. Matching Result Based on SAR Dataset

According to the changes of angle and scale, three kinds of image transformations are tested: 1. Scale, 2. Rotation, 3. Rotation scale conversion (scale and rotation exist simultaneously). In this experiment, the scale ratio is 0.9 and the rotation angle is 5.0 degrees.

**Figure 12:**
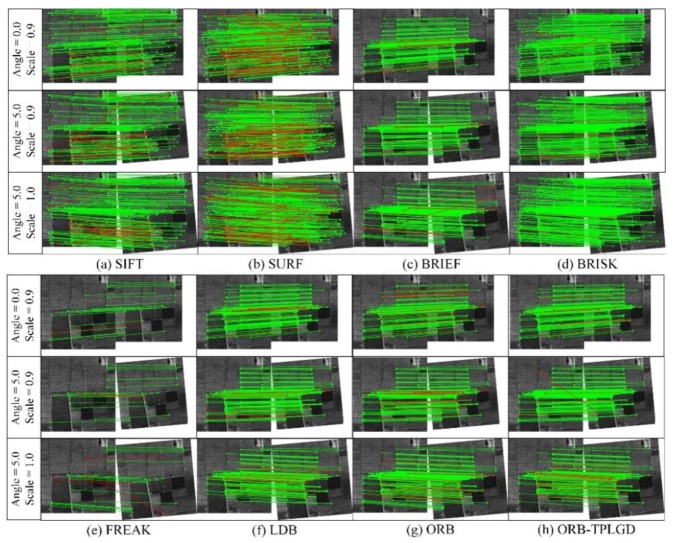
Matching effect based on the selected SAR images. (**a**) SIFT, (**b**) SURF, (**c**) BRIEF, (**d**) BRISK, (**e**) FREAK, (**f**) LDB, (**g**) ORB, (**h**) ORB-TPLGD.

A total of 100 SAR images are used for this experiment. This paper shows the matching effect of several randomly selected SAR images in Figure 12. The point pairs connected by green lines are considered to be the correct matching point pairs, while the point pairs connected by red lines are considered to be the wrong matching point pairs. The difference in the number of matching points can be seen from the results shown in Figure 12. The SIFT, SURF, and BRISK algorithms are capable of extracting a large number of feature points, and the number of the pairs of points considered to be matched is relatively large. The BRIEF, FREAK, LDB, ORB, and our improved ORB algorithms extract fewer feature points and have fewer pairs of points considered to be matched. The matching points extracted by SIFT and BRISK have fewer mismatches, so their matching precision is higher. Although SURF can extract a large number of matching points, there are many mismatches, which will reduce the matching precision. BRIEF, LDB, ORB, and our improved ORB algorithms use the same feature point extraction method, the number of feature points extracted is roughly the same.

#### 4.6.2. Matching Precision and Recall Based on SAR Dataset

In terms of matching precision, our improved algorithm has a relatively smaller number of wrong matching points than BRIEF, LDB, and ORB. The average of the matching precision and recall is recorded in Table 8.

According to the data in Table 8, SIFT is the most stable and the highest. For most images, the other algorithms cannot transcend SIFT. Although SURF is also based on floating-point feature description like SIFT, its matching precision is almost the lowest. Our improved ORB algorithm increases the length of the feature description and combining the local gray difference information to enhance its discriminating ability. The average precision of SIFT, SURF, BRIEF, BRISK, FREAK, LDB, ORB, and our improved ORB respectively is 95.275%, 84.473%, 93.520%, 96.663%, 93.194%, 89.963%, 94.109%, and 95.704%. Our improved ORB algorithm is improved by 2.481%, 2.828%, 4.462%, and 1.720% respectively compared with BRIEF, FREAK, LDB, and ORB.

In terms of recall recalls of SIFT, SURF algorithms are relatively high. SIFT, SURF, BRISK, and FREAK algorithms adopt respective feature point extraction methods and feature point description methods. ORB, LDB, FREAK, BRISK, BRIEF, and our improved ORB algorithms adopt the same feature point extraction method, causing the recall to be more dependent on the feature point description method. According to the data in Table 8, the average recall of SIFT, SURF, BRIEF, BRISK, FREAK, LDB, ORB, and our improved ORB respectively is 63.036%, 67.412%, 22.973%, 39.705%, 23.976%, 26.119%, 25.071%, and 27.119%. Our improved ORB algorithm is improved by 4.415%, 3.143%, 1.000%, and 2.048% respectively compared with BRIEF, FREAK, LDB, and ORB.

**Figure 13:**
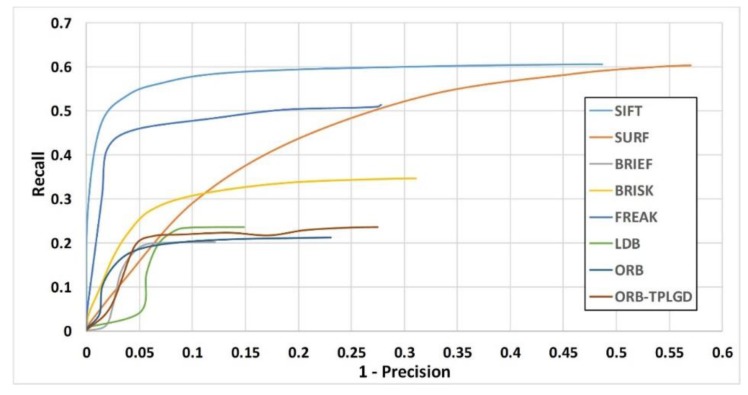
Matching effect based on the selected SAR images.

To examine the distinctiveness of binary descriptors we plot the recall versus 1-precision curves. We conduct experiments on 10 pairs of SAR images. The threshold value of distance ratio is varied to obtain the curve of the average recall versus average 1-precision. Figure 13 shows the recall versus 1-precision curves for SAR image pairs. Results show that ORB-TPLGD proposed in this paper outperforms the original ORB descriptor.

#### 4.6.3. Matching RMSE Based on SAR Dataset

**Figure 14:**
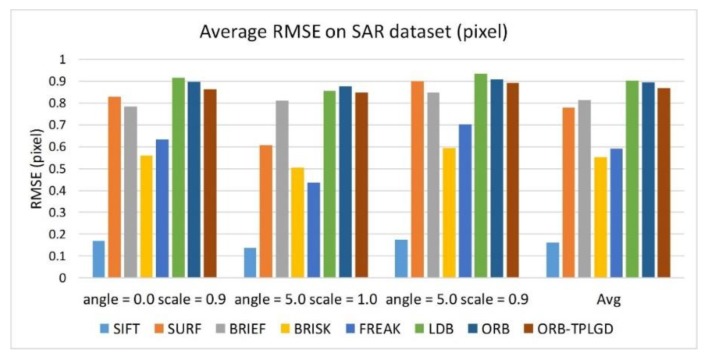
Average RMSE of test algorithms in the case of these three image transformations.

Figure 14 shows the RMSE value of each algorithm when testing the SAR dataset. Through RMSE in Table 9, we can see that SIFT algorithm has the highest positioning accuracy. Algorithms based on binary descriptions generally have lower positioning accuracy than SIFT algorithm. The algorithm proposed in this paper can get the current level of binary feature algorithm and has a certain improvement over the ORB algorithm.

#### 4.6.4. Matching Time Based on SAR Dataset

**Figure 15:**
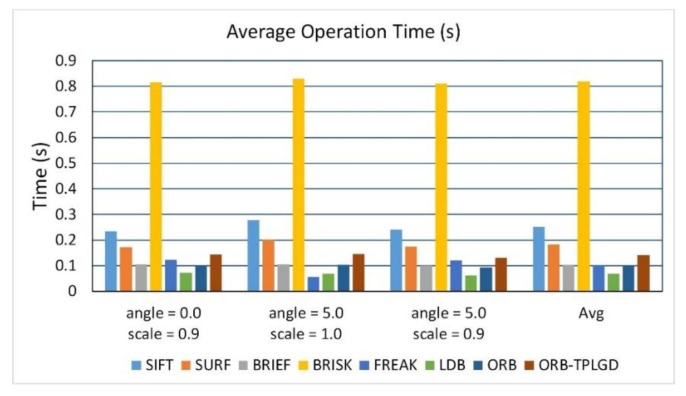
Average time required of test algorithms in the case of these three image transformations.

Figure 15 shows the average time required of test algorithms when testing the SAR dataset. Table 10 calculates the average matching time of the tested algorithms. According to Figure 15 and Table 10, the BRISK algorithm takes the longest time, followed by the SIFT algorithm. Other algorithms based on binary feature description take much less time than SIFT and SURF algorithms. This algorithm is slightly slower than the original ORB algorithm.

### 4.7. Statistical Analysis

In this section, we conduct statistical analysis to test whether the inputs create significant differences for the output. The independent variables include eight algorithms to be tested and the images to be tested. In the experimental part, we match the SAR images under three different transformations. The main performance metrics includes matching precision, recall rate, RMSE and matching time. We carried out two-way ANOVA, and we calculated the results of the above four metrics in the Table 11.

As shown in Table 11, the ‘Model’ is the test of variance analysis model used. For the above four metrics used, F-ratio value of ‘Model’ is 43.243, 275.752, 200.969, and 1265.541 respectively, and the corresponding P-values is less than 0.05. Therefore, the model used is statistically significant, and it can be used to judge whether the coefficient number in the model has statistical significance. The ‘Method’ and ‘Group’ in Table 11 represent the test method and image grouping respectively. For matching accuracy, recall, and RMSE, P-values of method and group are less than 0.05, which is statistically significant. It can be concluded that matching methods and input images have significant differences in matching precision, recall and RMSE. The analysis of variance of matching time shows that the P-value of ‘Method’ is less than 0.05, which shows that different methods have significant impact on matching time. The P-value of ‘Group’ is 0.092, which is greater than 0.05, indicating that different groups have no significant impact on the matching time.

Next step, we judge the difference between the two groups by pairwise test. Because the sample size of each group is equal, we use Tukey test method for multiple comparative analysis. The results of the multiple comparisons are as follows in Table 12 and Table 13.

Through the analysis of multiple comparison results of Tukey methods in Table 12 and Table 13, the following situations can be found.

In the aspect of matching precision, respectively compared with the ORB-TPLGD algorithm in pairs, the P-values of other algorithms is less than 0.05 except that SIFT and BRISK algorithm is 0.222 and 0.999. It shows that the ORB-TPLGD algorithm has significant difference with other algorithms except SIFT and BRISK algorithm.

In matching recall, respectively compared with ORB-TPLGD algorithm in pairs, the P-values of other algorithms is less than 0.05, which shows that ORB-TPLGD algorithm has significant difference with all other algorithms.

In terms of RMSE, respectively compared with the ORB-TPLGD algorithm in pairs, the P-values of other algorithms is less than 0.05 except that SIFT and BRIEF algorithm is 0.834 and 1.000. It shows that the ORB-TPLGD algorithm has significant difference with other algorithms except SIFT and BRIEF algorithm.

In matching time, respectively compared with the ORB-TPLGD algorithm in pairs, the P-values of other algorithms are less than 0.05 except that BRIEF, FREAK, and ORB are 0.965, 0.768, and 0.806, respectively. It shows that the ORB-TPLGD algorithm has significant differences with other algorithms except for BRIEF, FREAK, and ORB algorithms.

## 5. Discussion

Although the SIFT algorithm has the greatest stable and excellent matching effect, its calculation time is also the longest. SIFT and SURF algorithm have relatively high computational complexity, so the time they required is much longer than that of BRIEF, FREAK, LDB, ORB, and our improved ORB algorithms. This is mainly because SIFT algorithm constructs the scale pyramid on the feature point extraction stage. Moreover, SIFT algorithm extracts more feature points while a 128-dimensional floating-point description vector must be calculated for each feature point. The computational complexity is high. BRIEF is the most efficient followed by FREAK, ORB, and our improved ORB algorithm because that BRIEF is only required to perform the Hamming distance calculation between binary strings through simple exclusive or operation. However, BRIEF cannot suppress noise and has no rotation invariance. If the rotation exceeds a certain angle, the matching precision will decrease rapidly. Based on BRIEF, ORB reduce noise by filtering image and adds the main direction of feature points by calculating image moments. As a result, the operation time of ORB is slightly higher than BRIEF. The ORB-TPLGD algorithm proposed in this paper is based on the original ORB algorithm. A binary string coding process is carried out based on the three-patch groups comparison combining local gray difference. Although matching precision is improved, the running speed is reduced. Therefore, the computation speed of our improved ORB algorithm is a little smaller than that of BRIEF, FREAK, LDB, and ORB, while significantly higher than that of SIFT, SURF, and BRISK. BRISK is the slowest one among all tested binary feature description based matching algorithms even slower than SIFT and SURF. Through our experiments, we can find that the BRISK algorithm can extract a higher number of feature points than the SIFT algorithm, so it takes a lot of time. LDB is the second slowest because of its comparisons of mean intensity, horizontal gradient and vertical gradient in grids of with 2×2, 3×3, or 4×4 size. In general, the traditional feature description algorithm based on manual annotation, especially the algorithm based on binary feature description, has certain advantages in terms of calculation speed and calculation cost, which can be easily ported to portable devices.

## 6. Conclusions

In this paper, an improved ORB algorithm ORB using three-patch and local gray difference (ORB-TPLGD) is proposed. Based on the original ORB algorithm, this paper focuses on the feature point description process and improves the related methods. We mainly make two contributions: (i) a three-pixel patch groups comparison method is adopted to generate binary string instead of the pixel patch pairs comparison method used in original ORB algorithm. In each patches group, the gray value of the main patch is compared with that of the other two companion patches to determine the value of the corresponding bit of the final binary description. By this method, visual information with more spatial support is combined to generate feature description, which further reduces the sensitivity of feature description to noise. (ii) In our improved ORB algorithm, local gray difference information ignored by the original ORB algorithm is utilized to produce another binary string by a specific threshold. The final feature description of the feature point can be constructed by connecting above two binary strings to enhance its discrimination ability. In experiment section, our improved ORB algorithm is tested on Oxford dataset and SAR images dataset. Compared with SURF, BRIEF, FREAK, LDB, and ORB, our algorithm can achieve better matching performance in matching precision. In summary, our proposed ORB algorithm can achieve the state of-the-art performance.

## Figures and Tables

**Table 1:** Mean scores on the LFW, image-restricted training benchmark using Euclidean similarities [44].

Method	Original Images		Funneled		Alignment	
Descriptor	Euclidian	SQRT	Euclidian	SQRT	Euclidian	SQRT
LBP	0.6649	0.6616	0.6767	0.6782	0.6824	0.6790
TPLBP	0.6713	0.6678	0.6875	0.6890	0.6926	0.6897
FPLBP	0.6627	0.6572	0.6865	0.6820	0.6818	0.6746

**Table 2:** Test parameters, test methods, and parameter settings used in the experiment.

Method	Detection	Description	Matching	Description Size	Error Threshold	Optimization
SIFT	DOG	SIFT	Euclidean	128-dimensional	3 pixels	RANSAC
SURF	Hessian	SURF	Euclidean	64-dimensional	3 pixels	RANSAC
BRIEF	FAST	BRIEF	Hamming	32 bytes	3 pixels	RANSAC
BRISK	BRISK	BRISK	Hamming	64 bytes	3 pixels	RANSAC
FREAK	Harris	FREAK	Hamming	64 bytes	3 pixels	RANSAC
LDB	FAST	LDB	Hamming	32 bytes	3 pixels	RANSAC
ORB	FAST	ORB	Hamming	32 bytes	3 pixels	RANSAC
ORB-TPLGD	FAST	ORB-TPLGD	Hamming	64 bytes	3 pixels	RANSAC

**Table 3:** Matching precision based on Oxford dataset with different descriptor size or patch size (%).

	Size	Wall	Trees	Bikes	Leuven	Ubc	Boat	Average
Descriptor Size	4	83.134	35.945	82.532	67.370	97.616	44.028	68.437
	8	83.770	43.140	84.042	72.786	97.462	67.024	74.704
	16	85.233	45.614	84.514	71.885	98.052	68.862	75.693
	32	84.352	50.316	88.491	69.857	97.533	73.534	77.347
	64	83.990	49.600	86.459	72.066	97.431	75.964	77.585
	128	85.059	50.333	85.336	72.482	97.263	75.099	77.595
Patch Size	5×5	84.380	48.030	88.087	72.279	97.069	75.476	77.554
	7×7	83.990	49.600	86.459	72.066	97.431	75.964	77.585
	9×9	84.411	46.465	85.949	71.432	97.049	74.888	76.699
	11×11	86.099	47.394	80.668	72.860	97.990	76.385	76.899
	13×13	85.903	45.970	85.912	73.726	97.975	77.896	77.897
	15×15	84.461	47.122	87.314	71.663	98.675	78.717	77.992
	17×17	83.964	50.774	86.701	71.018	98.090	78.919	78.244

**Table 4:** Average matching precision based on Oxford dataset (%).

Method	Wall	Trees	Bikes	Leuven	Ubc	Boat	Average
SIFT	75.6656	73.1389	75.6656	90.1414	97.6497	83.6288	82.6483
SURF	56.5501	57.1606	56.5501	81.0738	81.4300	58.6993	65.2440
BRIEF	66.2154	64.6260	66.2154	83.6225	95.9177	60.5319	72.8548
BRISK	73.8393	61.2646	73.8393	93.7248	94.1188	74.9718	78.6264
FREAK	63.7331	62.2644	63.7331	81.5750	96.2506	74.2816	73.6396
LDB	59.1899	65.3837	59.1899	84.8628	96.3331	60.6045	70.9273
ORB	66.6433	68.9906	66.6433	84.3298	96.5113	77.8656	76.8307
ORB-TPLGD	67.5718	69.5843	67.5718	87.0085	96.7416	79.4594	77.9896

**Table 5:** Average matching recall based on Oxford dataset (%).

Method	Wall	Trees	Bikes	Leuven	Ubc	Boat	Average
SIFT	43.846	15.825	38.451	62.611	45.260	23.263	38.209
SURF	33.929	24.332	55.376	62.949	57.597	27.197	43.563
BRIEF	22.551	29.195	23.979	24.919	29.979	12.653	23.879
BRISK	18.219	14.853	51.324	41.308	37.627	12.948	29.380
FREAK	28.531	21.880	66.595	51.218	51.973	16.626	39.470
LDB	23.486	35.756	23.668	32.242	35.796	14.081	27.505
ORB	18.053	25.776	24.971	25.122	33.437	15.432	23.798
ORB-TPLGD	19.928	26.471	26.371	25.947	34.153	16.391	24.877

**Table 6:** Average matching RMSE based on Oxford dataset (pixel).

Method	Wall	Trees	Bikes	Leuven	Ubc	Boat	Average
SIFT	1.075	1.284	0.899	0.518	0.591	0.803	0.862
SURF	1.302	1.289	1.037	0.717	0.68	1.24	1.044
BRIEF	1.314	1.36	1.139	1.014	0.264	1.362	1.075
BRISK	1.257	1.33	1.192	0.738	0.736	1.12	1.062
FREAK	1.088	1.357	1.36	0.958	0.415	1.028	1.034
LDB	1.137	1.378	1.287	1.064	0.332	1.213	1.069
ORB	1.045	1.439	1.303	1.101	0.313	1.098	1.05
ORB-TPLGD	1.019	1.497	1.264	1.09	0.312	1.182	1.061

**Table 7:** Average matching time based on Oxford dataset (s).

Method	Wall	Trees	Bikes	Leuven	Ubc	Boat	Average
SIFT	7.638	8.176	1.462	1.603	3.064	4.267	4.368
SURF	4.650	3.967	1.797	1.272	2.368	2.500	2.759
BRIEF	0.488	0.586	0.214	0.219	0.328	0.381	0.369
BRISK	13.023	12.989	1.341	1.727	4.821	6.058	6.660
FREAK	0.494	0.596	0.244	0.222	0.347	0.408	0.385
LDB	1.128	1.243	0.844	0.855	0.963	1.026	1.010
ORB	0.589	0.703	0.337	0.308	0.417	0.485	0.473
ORB-TPLGD	0.734	0.872	0.420	0.384	0.646	0.608	0.611

**Table 8:** Average matching precision and recall based on SAR dataset (%).

	Angle = 0.0 Scale = 0.9		Angle = 5.0 Scale = 1.0		Angle = 5.0 Scale = 0.9		Average	
Method	Precision	Recall	Precision	Recall	Precision	Recall	Precision	Recall
SIFT	95.210	66.127	96.229	61.842	94.385	61.140	95.275	63.036
SURF	84.625	72.495	87.142	65.990	81.650	63.752	84.473	67.412
BRIEF	93.905	23.712	93.923	23.328	92.734	21.881	93.520	22.973
BRISK	96.977	37.797	97.304	44.936	95.708	36.381	96.663	39.705
FREAK	93.216	21.915	94.886	28.886	91.482	21.127	93.194	23.976
LDB	87.543	28.682	93.856	29.429	88.491	20.246	89.963	26.119
ORB	94.001	24.467	94.243	28.251	94.081	22.494	94.109	25.071
ORB-TPLGD	95.151	26.299	95.891	31.040	96.071	24.018	95.704	27.119

**Table 9:** Average matching RMSE of test image matching algorithms for SAR dataset (pixel).

Method	Angle = 0.0 Scale = 0.9	Angle = 5.0 Scale = 1.0	Angle = 5.0 Scale = 0.9	Average
SIFT	0.17	0.139	0.176	0.162
SURF	0.828	0.607	0.899	0.778
BRIEF	0.784	0.81	0.848	0.814
BRISK	0.559	0.505	0.593	0.552
FREAK	0.633	0.436	0.702	0.59
LDB	0.916	0.856	0.935	0.902
ORB	0.898	0.876	0.907	0.894
ORB-TPLGD	0.864	0.848	0.893	0.868

**Table 10:** Average matching time of test image matching algorithms for SAR dataset (s).

Method	Angle = 0.0 Scale = 0.9	Angle = 5.0 Scale = 1.0	Angle = 5.0 Scale = 0.9	Average
SIFT	0.234	0.277	0.240	0.250
SURF	0.172	0.202	0.175	0.183
BRIEF	0.105	0.107	0.099	0.104
BRISK	0.814	0.829	0.812	0.818
FREAK	0.123	0.057	0.120	0.100
LDB	0.074	0.068	0.062	0.068
ORB	0.099	0.104	0.094	0.099
ORB-TPLGD	0.144	0.146	0.132	0.140

**Table 11:** Two-way ANOVA for precision, recall, RMSE, and matching time.

Output	Source	Sum of Square	Degrees of Freedom	Mean Square	F-Ratio	P-Value
Precision	Model	2187.614	9	243.068	43.243	0.000
	Method	2037.921	7	291.132	51.794	0.000
	Group	149.693	2	74.846	13.316	0.000
	Error	618.303	110	5.621		
	Total	1039051.318	120			
Recall	Model	34933.154	9	3881.462	275.752	0.000
	Method	34275.051	7	4896.436	347.859	0.000
	Group	658.103	2	329.051	23.377	0.000
	Error	1548.350	110	14.076		
	Total	201249.612	120			
RMSE	Model	6.992	9	0.777	200.969	0.000
	Method	6.706	7	0.958	247.803	0.000
	Group	0.286	2	0.143	37.048	0.000
	Error	0.425	110	0.004		
	Total	66.492	120			
Time	Model	15979487.255	9	1775498.584	1265.541	0.000
	Method	15972653.737	7	2281807.677	1626.428	0.000
	Group	6833.517	2	3416.759	2.435	0.092
	Error	154325.173	110	1402.956		
	Total	24569866.561	120			

**Table 12:** Tukey test result for precision.

					95% Confidence Interval	
Method	Method	Average Difference	Standard Error	Significance	Lower Limit	Upper Limit
SIFT	BRISK	−1.65553182	0.865712842	0.546	−4.33066635	1.01960271
	FREAK	1.37694041	0.865712842	0.755	−1.29819412	4.05207494
	LDB	5.13483683	0.865712842	0.000	2.45970230	7.80997136
	SURF	11.42320018	0.865712842	0.000	8.74806564	14.09833471
	BRIEF	1.33043738	0.865712842	0.786	−1.34469715	4.00557191
	ORB	0.60079804	0.865712842	0.997	−2.07433650	3.27593257
	ORB-TPLGD	−2.13292593	0.865712842	0.222	−4.80806047	0.54220860
SURF	SIFT	−11.42320018	0.865712842	0.000	−14.09833471	−8.74806564
	BRISK	−13.07873199	0.865712842	0.000	−15.75386653	−10.40359746
	FREAK	−10.04625977	0.865712842	0.000	−12.72139430	−7.37112524
	LDB	−6.28836334	0.865712842	0.000	−8.96349787	−3.61322881
	BRIEF	−10.09276280	0.865712842	0.000	−12.76789733	−7.41762826
	ORB	−10.82240214	0.865712842	0.000	−13.49753667	−8.14726761
	ORB-TPLGD	−13.55612611	0.865712842	0.000	−16.23126064	−10.88099158
BRIEF	SIFT	−1.33043738	0.865712842	0.786	−4.00557191	1.34469715
	BRISK	−2.98596920	0.865712842	0.018	−5.66110373	−0.31083467
	FREAK	0.04650303	0.865712842	1.000	−2.62863150	2.72163756
	LDB	3.80439945	0.865712842	0.001	1.12926492	6.47953398
	SURF	10.09276280	0.865712842	0.000	7.41762826	12.76789733
	ORB	−0.72963934	0.865712842	0.990	−3.40477388	1.94549519
	ORB-TPLGD	−3.46336331	0.865712842	0.003	−6.13849785	−0.78822878
BRISK	SIFT	1.65553182	0.865712842	0.546	−1.01960271	4.33066635
	FREAK	3.03247223	0.865712842	0.015	0.35733770	5.70760676
	LDB	6.79036865	0.865712842	0.000	4.11523412	9.46550318
	SURF	13.07873199	0.865712842	0.000	10.40359746	15.75386653
	BRIEF	2.98596920	0.865712842	0.018	0.31083467	5.66110373
	ORB	2.25632985	0.865712842	0.165	−0.41880468	4.93146439
	ORB-TPLGD	−0.47739412	0.865712842	0.999	−3.15252865	2.19774042
FREAK	SIFT	−1.37694041	0.865712842	0.755	−4.05207494	1.29819412
	BRISK	−3.03247223	0.865712842	0.015	−5.70760676	−0.35733770
	LDB	3.75789642	0.865712842	0.001	1.08276189	6.43303096
	SURF	10.04625977	0.865712842	0.000	7.37112524	12.72139430
	BRIEF	−0.04650303	0.865712842	1.000	−2.72163756	2.62863150
	ORB	−0.77614237	0.865712842	0.986	−3.45127691	1.89899216
	ORB-TPLGD	−3.50986634	0.865712842	0.002	−6.18500088	−0.83473181
LDB	SIFT	−5.13483683	0.865712842	0.000	−7.80997136	−2.45970230
	BRISK	−6.79036865	0.865712842	0.000	−9.46550318	−4.11523412
	FREAK	−3.75789642	0.865712842	0.001	−6.43303096	−1.08276189
	SURF	6.28836334	0.865712842	0.000	3.61322881	8.96349787
	BRIEF	−3.80439945	0.865712842	0.001	−6.47953398	−1.12926492
	ORB	−4.53403880	0.865712842	0.000	−7.20917333	−1.85890427
	ORB-TPLGD	−7.26776277	0.865712842	0.000	−9.94289730	−4.59262824
ORB	SIFT	−0.60079804	0.865712842	0.997	−3.27593257	2.07433650
	BRISK	−2.25632985	0.865712842	0.165	−4.93146439	0.41880468
	FREAK	0.77614237	0.865712842	0.986	−1.89899216	3.45127691
	LDB	4.53403880	0.865712842	0.000	1.85890427	7.20917333
	SURF	10.82240214	0.865712842	0.000	8.14726761	13.49753667
	BRIEF	0.72963934	0.865712842	0.990	−1.94549519	3.40477388
	ORB-TPLGD	−2.73372397	0.865712842	0.041	−5.40885850	−0.05858944
ORB-TPLGD	SIFT	2.13292593	0.865712842	0.222	−0.54220860	4.80806047
	BRISK	0.47739412	0.865712842	0.999	−2.19774042	3.15252865
	FREAK	3.50986634	0.865712842	0.002	0.83473181	6.18500088
	LDB	7.26776277	0.865712842	0.000	4.59262824	9.94289730
	SURF	13.55612611	0.865712842	0.000	10.88099158	16.23126064
	BRIEF	3.46336331	0.865712842	0.003	0.78822878	6.13849785
	ORB	2.73372397	0.865712842	0.041	0.05858944	5.40885850

**Table 13:** Tukey test result of ORB-TPLGD part for recall, RMSE, and matching time.

					95% Confidence Interval	
Method	Method	Average Difference	Standard Error	Significance	Lower Limit	Upper Limit
ORB-TPLGD Recall	SIFT	−32.94057116	1.369958860	0.000	−35.65550807	−30.22563426
	SURF	−38.47078077	1.369958860	0.000	−41.18571767	−35.75584386
	BRIEF	5.92953077	1.369958860	0.000	3.21459386	8.64446767
	BRISK	−8.10979419	1.369958860	0.000	−10.82473109	−5.39485728
	FREAK	6.23592969	1.369958860	0.000	3.52099279	8.95086660
	LDB	2.97556157	1.369958860	0.032	0.26062467	5.69049848
	ORB	4.46683540	1.369958860	0.001	1.75189850	7.18177231
ORB-TPLGD RMSE	SIFT	0.66362698	0.022703694	0.000	0.59347042	0.73378354
	SURF	0.03282317	0.022703694	0.834	−0.03733339	0.10297974
	BRIEF	−0.00641205	0.022703694	1.000	−0.07656861	0.06374451
	BRISK	0.25355510	0.022703694	0.000	0.18339854	0.32371167
	FREAK	0.21858159	0.022703694	0.000	0.14842503	0.28873815
	LDB	−0.09820595	0.022703694	0.001	−0.16836251	−0.02804939
	ORB	−0.07616251	0.022703694	0.023	−0.14631907	−0.00600595
ORB-TPLGD Time	SIFT	−109.30440487	13.677017775	0.000	−151.56768291	−67.04112683
	SURF	−50.43650578	13.677017775	0.008	−92.69978382	−8.17322773
	BRIEF	14.41661464	13.677017775	0.965	−27.84666340	56.67989269
	BRISK	−1102.44706393	13.677017775	0.000	−1144.71034197	−1060.18378589
	FREAK	21.43998449	13.677017775	0.768	−20.82329355	63.70326254
	LDB	45.75413473	13.677017775	0.024	3.49085668	88.01741277
	ORB	20.51195562	13.677017775	0.806	−21.75132243	62.77523366
